# Supplementary material for: Uncovering the Multifaceted Role of PA2649 (nuoN) in Type III Secretion System and Other Virulence Production in Pseudomonas aeruginosa PAO1
Source: Microorganisms. 2025 Feb 11;13(2):392. doi: 10.3390/microorganisms13020392 (PMC11858028; doi:10.3390/microorganisms13020392)
Supplement: Supplementary file 1 [file microorganisms-13-00392-s001.zip › microorganisms-3404601-supplementary.pdf]

Additional file 1: Supplementary Information:

Uncovering the multifaceted role of *PA2649* (*nuoN*) in type III secretion system and other virulence production in *Paeruginosa aeruginosa* PAO1

Lin Chen [1,*,†], Yujie Si [1,†], Xue Han [1], Yue Xiao [1], Yidan Pan [1], Kangmin Duan [2] and Songzhe Fu [3,*]

[1] Key Laboratory of Resource Biology and Biotechnology in Western China, Ministry of Education, College of Life Science, Northwest University, Xi'an 710069, China; siyujie@stumail.nwu.edu.cn (Y.S.); hanxue@stumail.nwu.edu.cn (X.H.); xiaoyue@stumail.nwu.edu.cn (Y.X.); panyidan@stumail.nwu.edu.cn (Y.P.)

[2] Department of Medical Microbiology and Infectious Disease, Rady Faculty of Health Sciences, University of Manitoba, Winnipeg, MB R3E 0W2, Canada; kduan@nwu.edu.cn

[3] School of Medicine, Northwest University, Xi'an 710069, China

* Correspondence: chenlin@nwu.edu.cn (L.C.); fusongzhe@126.com (S.F.)

† These authors contributed equally to this work.

Supplementary Figureures and Tables

Table S1. Bacterial strains and plasmids used in this study

Strains or plasmid	Description	Reference or source
P. aeruginosa		
PAO1	Wild-type strain	This lab
Δ*PA2649*	*PA2649* knockout mutant of PAO1	This study
Δ*PA2649* (pAK-*nuoA-nuoN*)	Δ*PA2649* complemented strain; Cb^r	This study
Δ*rsmA*	*rsmA* knockout mutant of PAO1	This lab
Δ*PA2649* Δ*rsmA*	*PA2649* and *rsmA* knockout mutant of PAO1	This study
PAO1 (CTX-*exoS*)	PAO1 contains a CTX-*exoS* integration plasmid; Tc^r	This study
PAO1 (CTX-*rsmY*)	PAO1 contains a CTX-*rsmY* integration plasmid; Tc^r	This study
PAO1 (CTX-*rsmZ*)	PAO1 contains a CTX-*rsmZ* integration plasmid; Tc^r	This study
PAO1 (CTX-*rsmA*)	PAO1 contains a CTX-*rsmA* integration plasmid; Tc^r	This study
PAO1 (CTX-*exsC*)	PAO1 contains a CTX-*exsC* integration plasmid; Tc^r	This study
PAO1 (CTX-*exsD*)	PAO1 contains a CTX-*exsD* integration plasmid; Tc^r	This study
PAO1 (CTX-*PA1611*)	PAO1 contains a CTX-*PA1611* integration plasmid; Tc^r	This study
PAO1 (CTX-*retS*)	PAO1 contains a CTX-*retS* integration plasmid; Tc^r	This study
PAO1 (pKD-*hfq*)	PAO1 contains a pKD-*hfq* plasmid; Tmp^r	This study
PAO1 (pKD-*gacA*)	PAO1 contains a pKD-*gacA* plasmid; Tmp^r	This study
PAO1 (pKD-*gacS*)	PAO1 contains a pKD-*gacS* plasmid; Tmp^r	This study
Δ*PA2649* (CTX-*exoS*)	Δ*PA2649* contains a CTX-*exoS* integration plasmid; Tc^r	This study
Δ*PA2649* (CTX-*rsmY*)	Δ*PA2649* contains a CTX-*rsmY* integration plasmid; Tc^r	This study
Δ*PA2649* (CTX-*rsmZ*)	Δ*PA2649* contains a CTX-*rsmZ* integration plasmid; Tc^r	This study
Δ*PA2649* (CTX-*rsmA*)	Δ*PA2649* contains a CTX-*rsmA* integration plasmid; Tc^r	This study
Δ*PA2649* (CTX-*exsC*)	Δ*PA2649* contains a CTX-*exsC* integration plasmid; Tc^r	This study
Δ*PA2649* (CTX-*exsD*)	Δ*PA2649* contains a CTX-*exsD* integration plasmid; Tc^r	This study
Δ*PA2649* (CTX-*PA1611*)	Δ*PA2649* contains a CTX-*PA1611* integration plasmid; Tc^r	This study
Δ*PA2649* (CTX-*retS*)	Δ*PA2649* contains a CTX-*retS* integration plasmid; Tc^r	This study
Δ*PA2649* (pKD-*hfq*)	Δ*PA2649* contains a pKD-*hfq* plasmid; Tmp^r	This study
Δ*PA2649* (pKD-*gacA*)	Δ*PA2649* contains a pKD-*gacA* plasmid; Tmp^r	This study
Δ*PA2649* (pKD-*gacS*)	Δ*PA2649* contains a pKD-*gacS* plasmid; Tmp^r	This study
E.coli		
S17-1 λpir	*RP4-2 (Km::Tn7, Tc::Mu-1) pro-82 λpir recA1 endA1 thiE1 hsdR17 creC510*	
DH5α	*F^- φ80lacZ ΔM15 Δ(lacZYA-argF)U169 recA1 endA1 hsdR17(rk$^-$, mk$^+$)phoA supE44 thi-1 gyrA96 relA1 tonA*	Invitrogen
Plasmids		
CTX-*exoS*	Integration plasmid, CTX6.1 with a fragment of pKD-*exoS* containing *exoS* promoter region and *luxCDEAB* gene; Kan^r, Tmp^r, Tc^r	[1]
CTX-*rsmY*	Integration plasmid, CTX6.1 with a fragment of pKD-*rsmY* containing *rsmY* promoter region and *luxCDEAB* gene; Kan^r, Tmp^r, Tc^r	[1]
CTX-*rsmZ*	Integration plasmid, CTX6.1 with a fragment of pKD-*rsmZ* containing *rsmZ* promoter region and *luxCDEAB*	[1]

	gene; Kan^r, Tmp^r, Tc^r	
CTX-*exsC*	Integration plasmid, CTX6.1 with a fragment of pKD-*exsC* containing *exsC* promoter region and *luxCDEAB* gene; Kan^r, Tmp^r, Tc^r	[1]
CTX-*exsD*	Integration plasmid, CTX6.1 with a fragment of pKD-*exsD* containing *exsD* promoter region and *luxCDEAB* gene; Kan^r, Tmp^r, Tc^r	[1]
CTX-*PA1611*	Integration plasmid, CTX6.1 with a fragment of pKD-*PA1611* containing *PA1611* promoter region and *luxCDEAB* gene; Kan^r, Tmp^r, Tc^r	[1]
CTX-*retS*	Integration plasmid, CTX6.1 with a fragment of pKD-*retS* containing *retS* promoter region and *luxCDEAB* gene; Kan^r, Tmp^r, Tc^r	This lab
CTX-*rsmA*	Integration plasmid, CTX6.1 with a fragment of pKD-*rsmA* containing *rsmA* promoter region and *luxCDEAB* gene; Kan^r, Tmp^r, Tc^r	This lab
pKD-*hfq*	pMS402 containing *hfq* promoter region; Kan^r, Tmp^r	This lab
pKD-*gacA*	pMS402 containing *gacA* promoter region; Kan^r, Tmp^r	This lab
pKD-*gacS*	pMS402 containing *gacS* promoter region; Kan^r, Tmp^r	This lab
pAK1900	*E.coli-P.aeruginosa* shuttle cloning vector carrying plac upstream of MCS, Amp^r, Cb^r	[2]
pAK-*PA1611*	pAK1900 with the entire *PA1611* gene and the promoter region of *PA1611*; Amp^r, Cb^r	[1]
pAK-*ladS*	pAK1900 with the entire *ladS* gene and the promoter region of *ladS*; Amp^r, Cb^r	[1]
pAK-*rsmY*	pAK1900 with the entire *rsmY* gene and the promoter region of *rsmY*; Amp^r, Cb^r	[3]
pAK-*rsmZ*	pAK1900 with the entire *rsmZ* gene and the promoter region of *rsmZ*; Amp^r, Cb^r	[3]
pAK-*hfq*	pAK1900 with the entire *hfq* gene and the promoter region of *hfq*; Amp^r, Cb^r	This lab
pAK-*exsD*	pAK1900 with the entire *exsD* gene and the promoter region of *exsD*; Amp^r, Cb^r	This study
pAK-*gacA*	pAK1900 with the entire *gacA* gene and the promoter region of *gacA*; Amp^r, Cb^r	This study
pAK-*gacS*	pAK1900 with the entire *gacS* gene and the promoter region of *gacS*; Amp^r, Cb^r	This study
pAK-*PA2649*	pAK1900 with the entire *PA2649* gene and the promoter region of *PA2649*; Amp^r, Cb^r	This study
pAK-*PA2650*	pAK1900 with the entire *PA2650* gene and the promoter region of *PA2650*; Amp^r, Cb^r	This study
pAK-*PA2649-PA2650*	pAK1900 with the entire *PA2649* and *PA2650* gene and the promoter region of *PA2649* and *PA2650*; Amp^r, Cb^r	This study
pAK-*pro+PA2649*	pAK1900 with the entire *PA2649* and *nuo* operon promoter gene; Amp^r, Cb^r	This study
pAK-*PA2645-PA2650*	pAK1900 with the entire *PA26495, PA2646, PA2647, PA2648, PA2649* and *PA2650* gene and the promoter region of *PA26495, PA2646, PA2647, PA2648, PA2649* and *PA2650*; Amp^r, Cb^r	This study
pAK-*nuoA-nuoN*	pAK1900 with the entire *nuo* operon gene and the promoter region of *nuo* operon; Amp^r, Cb^r	This study

pEX-18Tc	Tc^R; *ori*T⁺ *sac*B⁺, gene replacement vector with MCS from pUC18	[4]
pEX-*PA2649*	*PA2649* knock out plasmid; Tc^r	This study

Table S1. Primers used in this study

Primer	Sequence(5′→3′)	Restriction site
PA2649-up1	ATGACCATGATTACGAATTCATGATTCTGCCCTGGCTAATCC	*Eco*R I
PA2649-down1	GGCAGGAGCGCGATGAAGTG	None
PA2649-up2	CACTTCATCGCGCTCCTGCCAATCCTCCAGCATTCCGGC	None
PA2649-down2	ACGGCCAGTGCCAAGCTTCAGGGCTCGTCGCTGTCAAC	*Hin*dIII
PA2649-up	AAGAGCTCACGAGCCGATCCT	*Sac* I
PA2649-down	CGCAAGCTTCCATTCGCAGT	*Hin*dIII
PA2649pro-up	TATAGGGCGAATTCGAGCTCTGGTCAGCGACGAGAACG	*Sac* I
PA2649pro-down	CCGAGGATCGGCTCGTATGCCTTGCTGCCAAGCAGG	None
PA2649-down3	ACACTATAGAATACTCAAGCTTCGCGACCATTCGCAGTCACG	*Hin*dIII
PA2645-2650-up	TATAGGGCGAATTCGAGCTCCTGGTCGCTGCCTATCACCTG	*Sac* I
PA2649-2650-up	TATAGGGCGAATTCGAGCTCCTTGCAGGACGGTAGTCG	*Sac* I
PA2645-50-down	ACACTATAGAATACTCAAGCTTGCGAACTGAACTTCGCCGCC	*Hin*dIII
PA2650-up	CGGGATCCGCTGCTGGAAATCCT	*Bam*HI
PA2650-down	CCCAAGCTTATGGACGGCGCTGT	*Hin*dIII
nuoA-nuoN-up1	ACTATAGGGCGAATTCGAGCTCGGAGCGACTACATCTACGCTGC	*Sac* I
nuoA-nuoN-down1	CCACGGTGAGGATGATGGTGACCAGAGC	None
nuoA-nuoN-up2	CACCATCATCCTCACCGTGGTCAAGGCCATCGTGGTGCTGCTC	None
gacS-up	CTAAGCTTGTTGAGCGTCAGGATCAGGCC	*Hin*dIII
gacS-down	CGGATCCATCGTGCCAGTATTCACGATC	*Bam*H I
exsD-up	TATAGGGCGAATTCGAGCTCAGCGGCTGCATGAGTAGAATC	*Sac* I
exsD-down	ACACTATAGAATACTCAAGCTTCCTTGGCCCAGGCGAGTGGC	*Hin*dIII
gacA-up	AATAAGCTTGGCCTCCGCATCGAACATG	*Hin*dIII
gacA-down	TATGGTACCGTGAGATGGCCGCACAGTAG	*Kpn* I

Note: Underlined sequences indicate restriction sites.

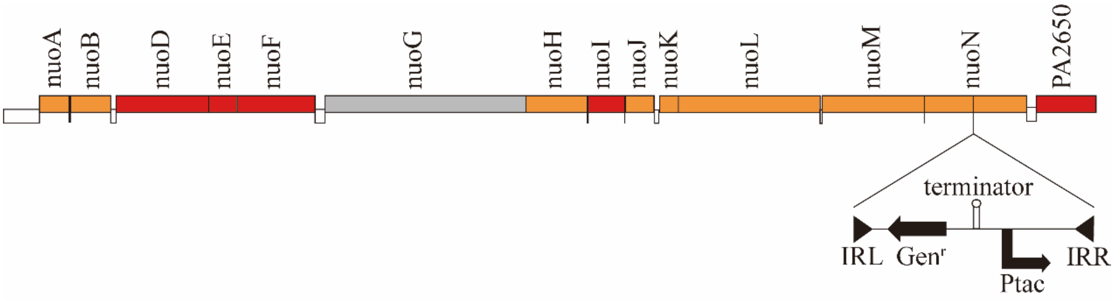

Figure S1. Schematic illustration of the pBT20 transposon insertion site within the *PA2649* gene.

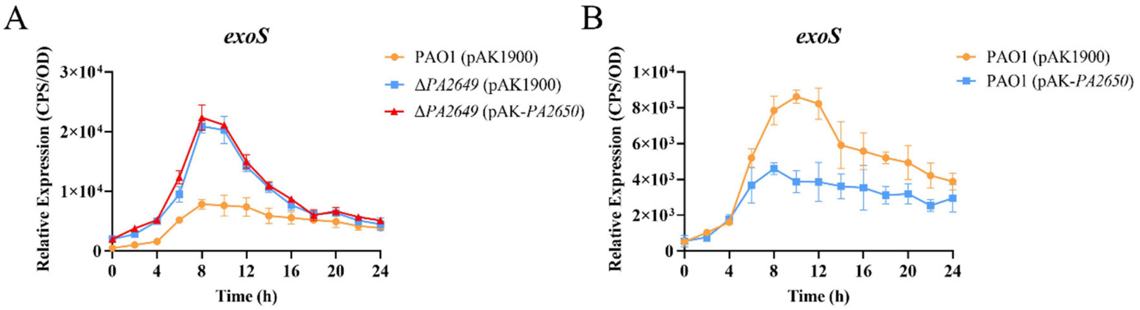

Figure S2. Effect of *PA2649* mutation and *PA2650* overexpression on *exoS* expression. (A) Expression levels of *exoS* were measured using a chromosomally integrated CTX-*exoS* reporter fusion in wild-type PAO1, the *PA2649* mutant, and a *PA2650* overexpression strain under T3SS-inducing conditions. (B) *exoS* expression in PAO1 carrying *PA2650* overexpression plasmids. Data represent the mean of three independent experiments, with error bars indicating standard deviations. Assays were conducted in triplicate. cps, counts per second.

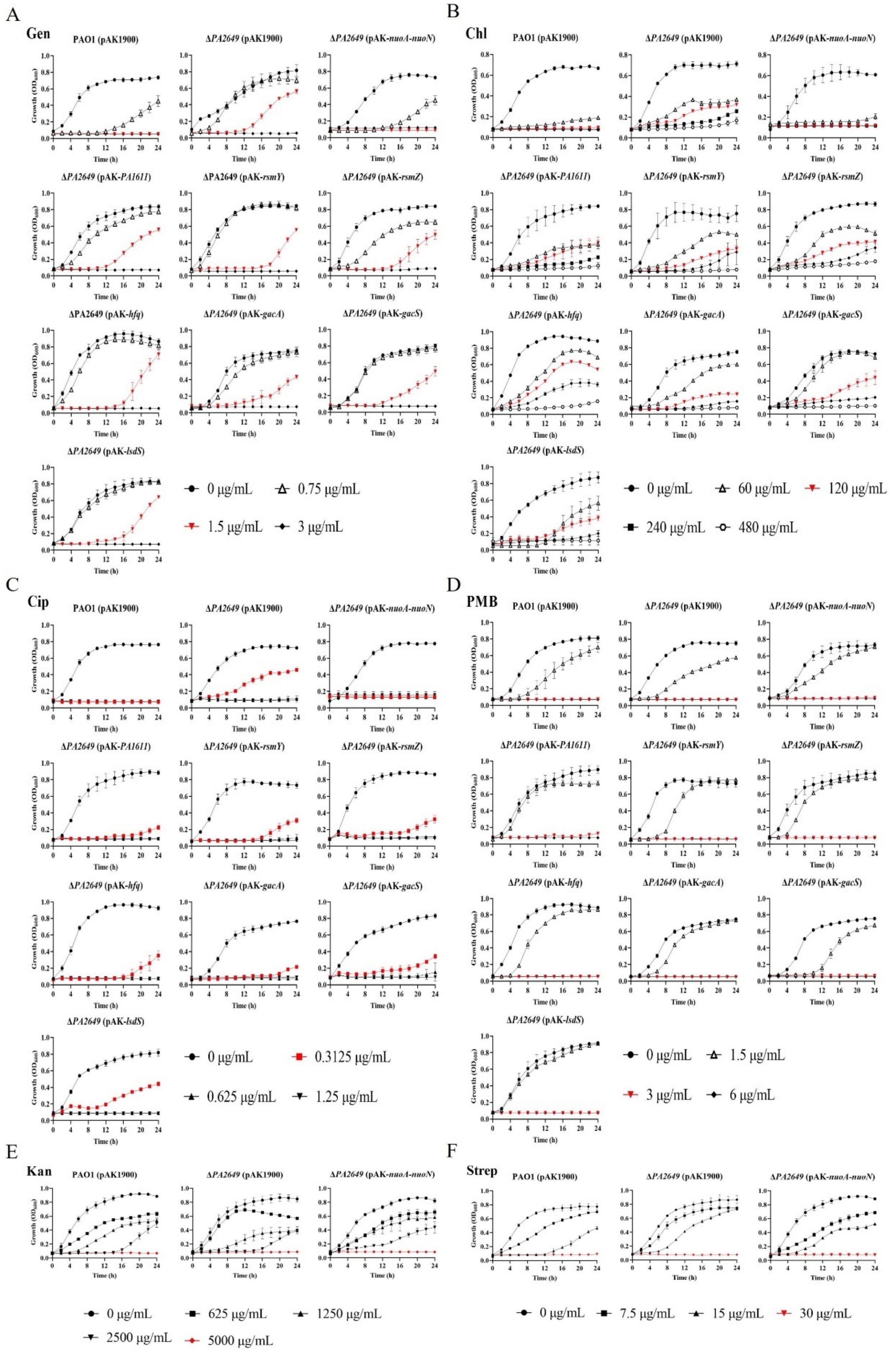

Figure S3. Antibiotic susceptibility profiles of PAO1, *PA2649* mutants, and overexpression strains for *PA1611*, *rsmY*, *rsmZ*, *hfq*, *gacA*, *gacS*, and *ladS*. Antibiotic susceptibility of wild-type PAO1, *PA2649* mutants, and strains overexpressing PA1611, *rsmY*, *rsmZ*, *hfq*, *gacA*, *gacS*, and *ladS* was assessed against (A) Gentamicin

(Gen), (B) Chloramphenicol (Chl), (C) Ciprofloxacin (Cip), (D) Polymyxin B (PMB), (E) Kanamycin (Kan), and (F) Streptomycin (Strep). Results reflect the mean of three independent experiments, with error bars indicating standard deviations.

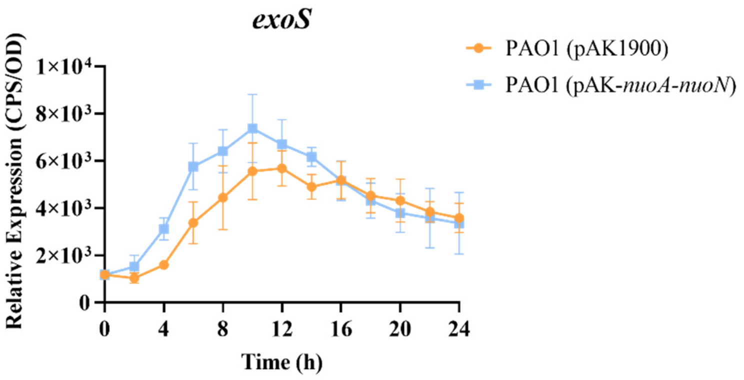

Figure S4. Effect of *nuo* operon overexpression on *exoS* expression in PAO1. Expression levels of *exoS* in wild-type PAO1 harboring plasmids overexpressing the *NUO* operon were measured using a chromosomally integrated CTX-*exoS* reporter fusion. Results represent the mean of three independent experiments, with error bars indicating standard deviations. Counts per second (cps) were used as the measurement unit.

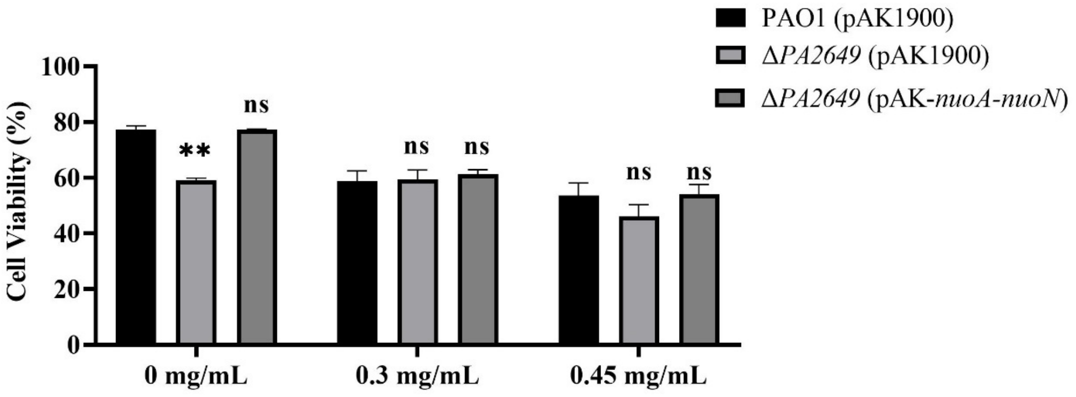

Figure S5. Cytotoxicity assay of *P. aeruginosa* strains in A549 cells with proteinase K treatment. A549 cells were infected with various *P. aeruginosa* strains for 3 hours, and cytotoxicity was assessed using the CCK-8 assay. Supernatants from each strain were treated with different concentrations of proteinase K to inactivate other proteins content. Data represent the mean of three independent experiments, with error bars showing standard deviations. Significance levels: ns (not significant, $p > 0.05$); *$p < 0.05$; **$p < 0.01$.

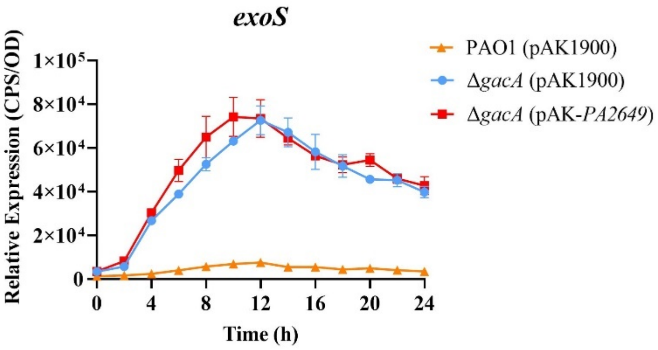

Figure S6. Expression of *exoS* in *gacA* mutants with overexpressed *PA2649*. *exoS* expression levels were measured in *gacA* mutant strains with overexpression of *PA2649* using a chromosomally integrated CTX-*exoS* reporter fusion. Results represent the average of three independent experiments, with error bars indicating standard deviations. Measurements are in counts per second (cps).

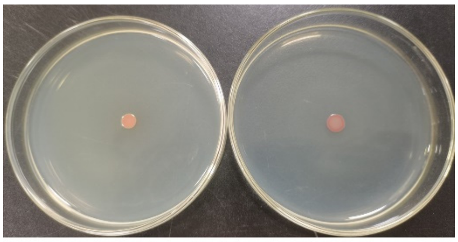

PAO1 ΔPA2649

Figure S7. Respiratory chain integrity assessment in *PA2649* mutant using TTC reduction assay. The TTC reduction assay was employed to evaluate respiratory chain integrity in the *PA2649* mutant. A red coloration indicates a complete and functional respiratory chain, while white denotes respiratory chain damage.

Reference

1. Kong, W.; Chen, L.; Zhao, J.; Shen, T.; Surette, M.G.; Shen, L.; Duan, K. Hybrid sensor kinase PA1611 in *Pseudomonas aeruginosa* regulates transitions between acute and chronic infection through direct interaction with RetS. *Molecular microbiology* **2013**, *88*, 784-797, doi:10.1111/mmi.12223.
2. Poole, K.; Neshat, S.; Krebes, K.; Heinrichs, D.E. Cloning and nucleotide sequence analysis of the ferripyoverdine receptor gene fpvA of *Pseudomonas aeruginosa*. *Journal of bacteriology* **1993**, *175*, 4597-4604, doi:10.1128/jb.175.15.4597-4604.1993.
3. Dadashi, M.; Chen, L.; Nasimian, A.; Ghavami, S.; Duan, K. Putative RNA Ligase RtcB Affects the Switch between T6SS and T3SS in *Pseudomonas aeruginosa*. *International journal of molecular sciences* **2021**, *22*, doi:10.3390/ijms222212561.
4. Hoang, T.T.; Karkhoff-Schweizer, R.R.; Kutchma, A.J.; Schweizer, H.P. A broad-host-range Flp-FRT recombination system for site-specific excision of chromosomally-located DNA sequences: application for isolation of unmarked *Pseudomonas aeruginosa* mutants. *Gene* **1998**, *212*, 77-86, doi:10.1016/s0378-1119(98)00130-9.
